# Leukæmia1Read before the Missouri Valley Veterinary Association, February, 1899.

**Published:** 1899-06

**Authors:** B. F. Kaupp

**Affiliations:** Kansas City, Mo.


### LEUKÆMIA[1].

By B. F. Kaupp, D.V.S.,
KANSAS CITY, MO.

Leucocythæmia, sometimes called leukæmia, is a disease which affects horses, cattle, hogs, and other animals, including man. Zuill says it has never been reported in a sheep or a goat.

The disease is characterized by a great and permanent increase in the number of white blood-corpuscles, by a diminution in the

[1] Read before the Missouri Valley Veterinary Association, February, 1899.

number of red blood-corpuscles, and by an enlargement of the lymphatic glands. The spleen is one of the organs most frequently affected. In the normal state there is found 1 white blood-corpuscle to 350 red in nearly all warm-blooded animals; according to Zuill, in the horse and dog there is one white blood-corpuscle to about 800 red. In leucocythæmia there may be one white corpuscle to 50, 25, or even 2 red.

The direct cause of this disease seems to be unknown. The primary lesions involve one of the blood-making organs—the spleen, lymphatic ganglions, or red marrow of the bones. The lymphatic glands most often affected are the ganglions of the head, neck, and shoulders, of the extremities, and of the abdominal and pectoral cavities; these glands become tumefied and hypertrophied. The disease may also involve the spleen, liver, kidneys, uterus, bladder, lungs, subcutaneous connective tissue, the serous membranes, mucous membranes, etc. Leukæmia infarcts consist of a diffused infiltration of the tissue by white corpuscles—an infiltration which surrounds the bloodvessels with a kind of whitish-gray membrane. The new lymphlike tissues are circumscribed tumors, the histological structure somewhat resembling that of lymphatic ganglion. The spleen becomes enlarged, sometimes to enormous dimensions; the enlargement uniform, the organ presenting practically the normal shape; the capsule often thickened; the cut surface smooth and of grayish or brownish-red color; the Malpighian corpuscles are usually enlarged and prominent; the trabecular tissue often thickened, and can be seen marking it as whitish lines. The enlargement of the spleen is mainly due to the increase of the splenic pulp. The enlarged lymphatic glands are grayish or reddish in color, and sometimes mottled in appearance on the cut surface; microscopically the enlarged glands show increase of the pulp and blocking of the lymph-paths.

Leukæmia appears to be more common in cattle than in other animals in this section of the country. Steel speaks of this disease occurring most often in female animals. The three cases I have had occasion to examine have occurred in cows, as follows:

Case I.—August 1, 1896. Subject, an aged cow, weighing, if in good condition, probably 1000 pounds. The animal had an unthrifty appearance. The whole carcass, when dressed, presented somewhat of a jaundiced condition. The liver had a light brown color and was considerably enlarged. The spleen was about five times the normal size, and, when cut through, the cut surface presented a brownish-black appearance. Malpighian corpuscles enlarged and plainly visible, about the size of millet-seeds, and lighter in color than the splenic pulp. Some of the lymphatic glands were enlarged, and when cut through presented apparently normal gland-tissue.

CASE II.—January 10, 1898. Subject, a seven-year-old native cow, weighing 900 pounds. Color: Red, with white spots; in poor condition, with staring coat and unthrifty appearance. Upon post-mortem the spleen was found to be enormously enlarged, three feet long, twelve inches wide, and four inches thick in the thickest portion. Malpighian corpuscles greatly enlarged and of whitish appearance. Lymphatic glands all over the body were greatly enlarged. The bronchial lymphatic glands varied in size from a hen's egg to a goose-egg. Some of the glands in the pelvic region as well as the lumbar lymphatic glands and others were the size of a hen's egg and larger.

Dr. S. Stewart reports a case of leucocythæmia in a cow, in the January number of the *American Veterinary Review*, in 1897, which case it was also my good fortune to examine. The post-mortem examination revealed general emaciation, with all the structures and viscera presenting the usual appearance of emaciated animals, with the exception of the lymphatic glands and the spleen. The lymphatic glands in all parts of the body were greatly enlarged, varying in size from a walnut to a goose-egg and larger, and seemed upon section to consist of hypertrophied glandular tissue. The spleen, which ordinarily would have weighed one and one-half pounds, weighed nineteen and one-half pounds, and measured thirty-two inches in length, nine inches in breadth, and three inches in the thickest portion. Upon section the Malpighian corpuscles presented themselves as nearly whitish bodies, many of them one-third to one-half inch in diameter. The remainder of the splenic pulp was normal in color and consistence. The result of the microscopical examination of the blood showed one white blood-corpuscle to twenty red. The prescapular lymphatic glands weighed three pound a piece, the bronchial glands one and one-half pounds, and the deep iliac two pounds. Some of the superficial lymphatic glands were sufficiently enlarged to be located upon ante-mortem examination. Dr. Stewart also reports a case of leucocythæmia in a St. Bernard dog.

Dr. T. W. Carnachan, of Kansas City, reports an interesting case in a steer, as follows: The subject of this case was a Western steer, about three years of age. Nothing unusual was presented by the animal before death, but on post-mortem examination the following well-defined lesions were presented to view. The spleen was very much enlarged in all its dimensions. The length was such as to cause it to cross the abdominal cavity, and then curl up and turn over past the centre of the diaphragm. It weighed almost a hundred pounds. The liver was enlarged and of a very pale color, the same condition applying to the heart. All the abdominal and thoracic lymphatic glands were very much enlarged and of the same color as the heart and liver. The flesh of the carcass was of a pale hue and very flabby in consistency. The animal was much thinner in flesh than the balance of the same bunch.

Dr. Carnachan also reports a case in a hog, as follows: The animal was killed in February, 1899. Not seeing it before death I can give no ante-mortem report, but on post-mortem I noticed it being much thinner than the rest of the bunch, but not enough to pronounce it emaciated. The skin was rough over the greater part of the body, which, when handled, was found to be a mass of small, nodular enlargements which contained pus. The skin was of a yellowish hue. The bladder was unusually large for such a small animal. The liver was enlarged and of a very pale color. The spleen was much enlarged; also the abdominal and thoracic lymphatic glands, especially those of the pelvic and sublumbar regions. These glands were as large as an egg, and pale in color. The adipose tissue was thin and watery in consistency. The animal was small and not many months old.

